# Omental Pseudocyst Following an Open Groin Surgery: A Cause for Concern

**DOI:** 10.7759/cureus.76743

**Published:** 2025-01-01

**Authors:** Vivek Peddakota, Manjunath Maruti Pol, Jagadeep Ajmera, Belmin BJ Winston Gysley

**Affiliations:** 1 General Surgery, All India Institute of Medical Sciences, New Delhi, IND

**Keywords:** abdominal pain, non-absorbable sutures, omental cyst, omental pseudocyst, open mesh hernioplasty, rare surgical complication, suture migration

## Abstract

Retained, non-absorbable sutures following surgical procedures can lead to rare complications, including pseudocyst formation. Here, we present a unique case of an omental pseudocyst caused by suture migration after an open inguinal hernia repair with mesh. A 31-year-old male with a history of right-sided open mesh hernioplasty performed two years prior presented with dull, aching abdominal pain persisting for six months. Clinical examination was unremarkable except for a right groin scar. Ultrasonography and contrast-enhanced computed tomography (CT) of the abdomen revealed a complex omental cyst and cholelithiasis. The patient underwent laparoscopic excision of the omental cyst and cholecystectomy. The excised cyst contained knotted, non-absorbable sutures and pus-like fluid, confirming the diagnosis of an omental pseudocyst caused by suture migration. This case highlights three key concerns regarding retained non-absorbable sutures: the potential for suture migration, pseudocysts formation, and challenges in diagnosing rare post-operative complications.

## Introduction

The omental cyst is a rare condition. It was first reported in 1852 by Gairdner [1]. The in-hospital incidence of combined omental and mesenteric cysts is one in 105,000 to 140,000 [2]. Omental cysts are three to 10 times rarer than mesenteric cysts [2]. Most of them are seen in children under 10 years old and are more common in females. Omental lesions can be classified into true omental cysts and pseudocysts. True omental cysts are lined by endothelium and contain rich lymphatic channels, whereas pseudocysts lack an epithelial lining. The true incidence of pseudo-omental cysts is unknown due to their rarity and asymptomatic nature. They are less frequently reported, possibly due to medicolegal implications. To our knowledge, there is no documented report in the English literature of a suture-causing omental pseudocyst following open inguinal hernia repair.

## Case presentation

A 31-year-old male presented to the surgical clinic with complaints of dull, aching abdominal pain in the periumbilical, right lumbar, right hypochondriac, and epigastric regions for the past six months. The pain was insidious in onset, ill-defined, and mild to moderately intense, with no radiation. The patient had undergone open mesh hernioplasty at a different hospital for a right-sided inguinal hernia two years earlier. He had consulted several doctors for abdominal pain and took medications before he was referred to our hospital. 

On examination, the patient was hemodynamically stable. The abdomen was soft, non-distended, and non-tender. There was no organomegaly or a palpable lump. A scar was present in the right inguinal region, suggestive of previous hernia surgery. There were no other significant systemic findings.

Investigations

**Figure 1:**
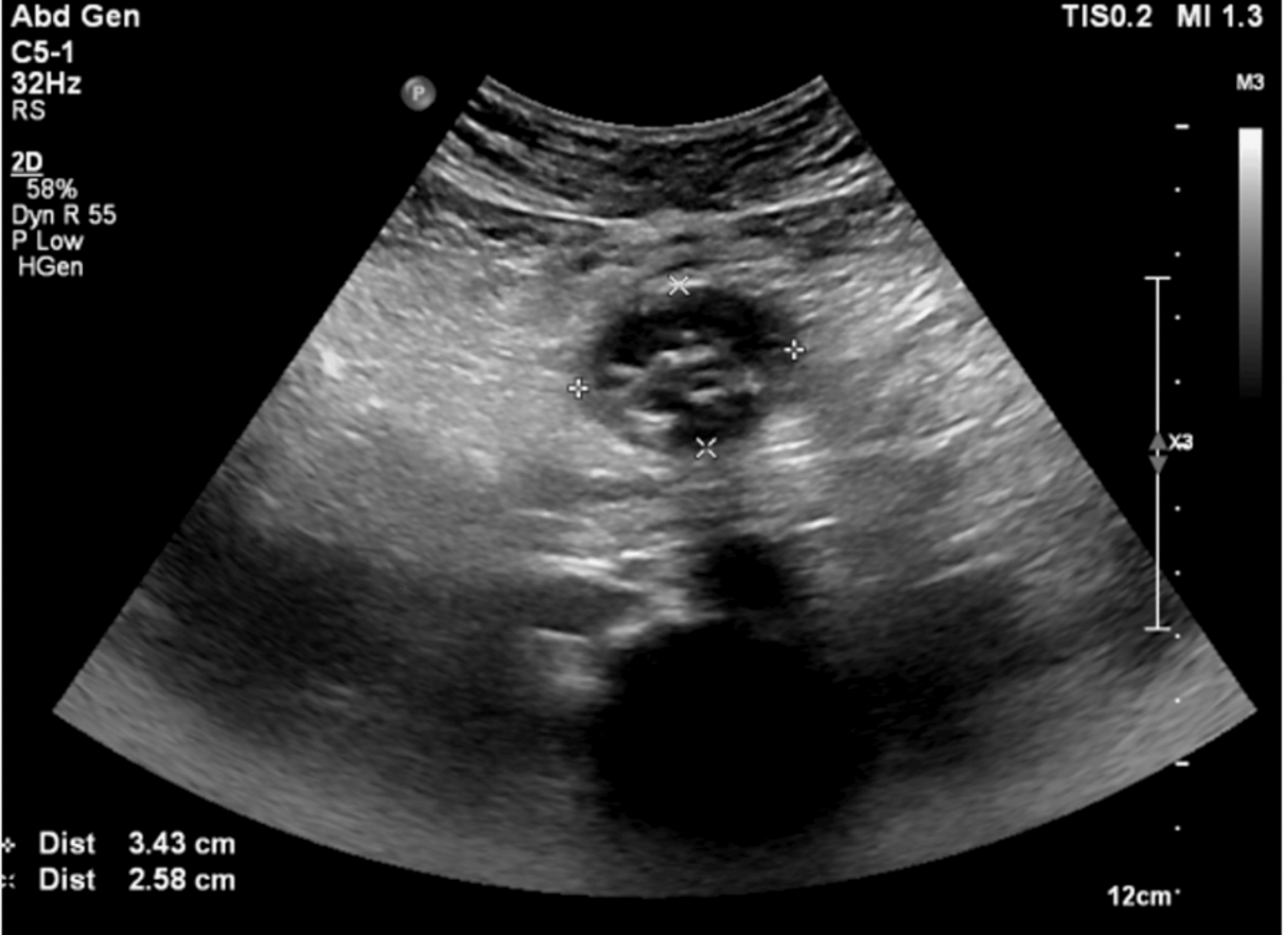
Ultrasonography of the abdomen showing multiple calculi in the gallbladder and a well-defined heteroechoic cystic area with thick walls and a few echogenic foci in the region of the omentum

**Figure 2:**
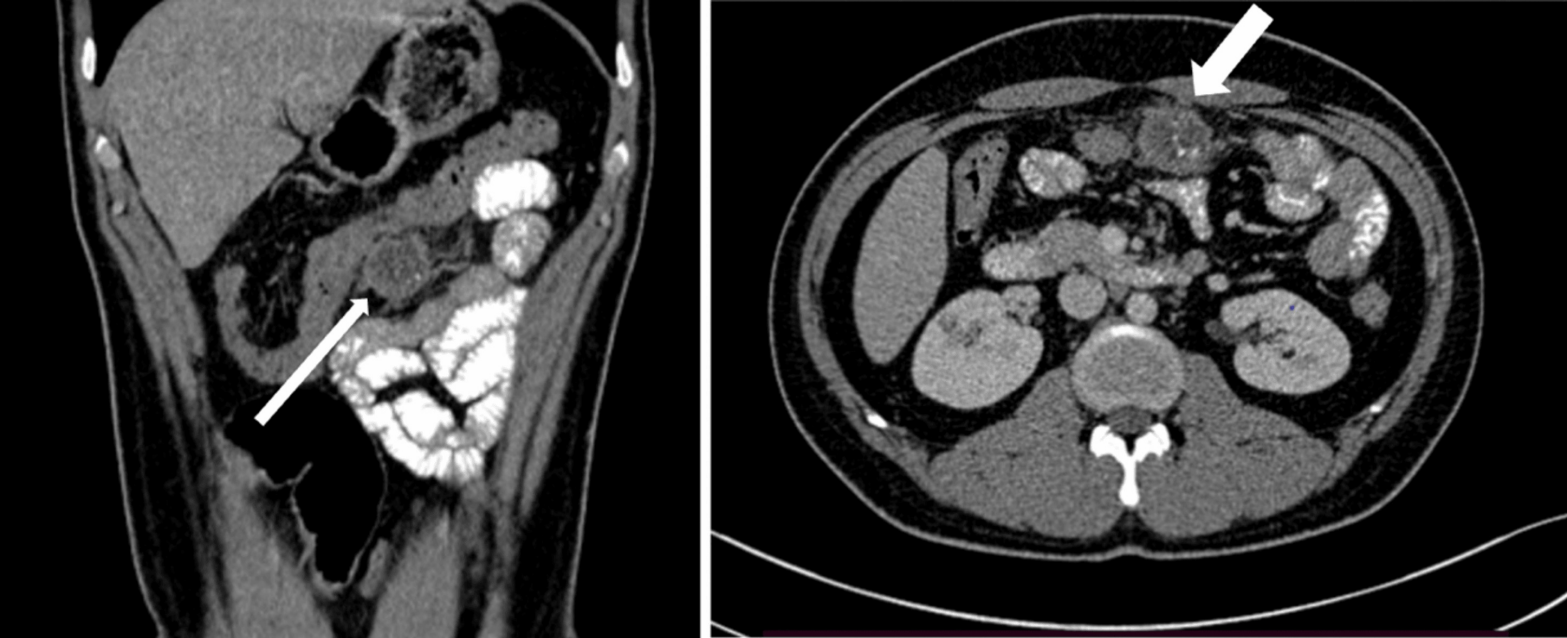
Computed tomography of the abdomen showing foci of calcification adjacent to the transverse colon, suggestive of a complex omental cyst or omental infarct CT, computed tomography

The patient's hemogram, liver function tests, and renal function tests were all within the normal range. Ultrasonography of the abdomen revealed a well-defined heteroechoic lesion (3.5x2.5 cm) in the region of the omentum, showing cystic areas with thick walls, a few echogenic foci, and multiple calculi in the gallbladder (Figure 1). The contrast-enhanced computed tomography (CT) scan of the abdomen showed a hypodense lesion measuring 3.2x2.4x2.4 cm, with foci of calcification adjacent to the transverse colon, suggestive of a complex omental cyst or omental infarct (Figure 2).

Treatment

**Figure 3:**
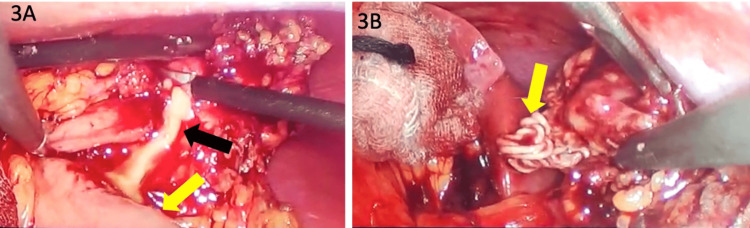
Intraoperative images of ruptured cyst containing pus and braided sutures

**Figure 4:**
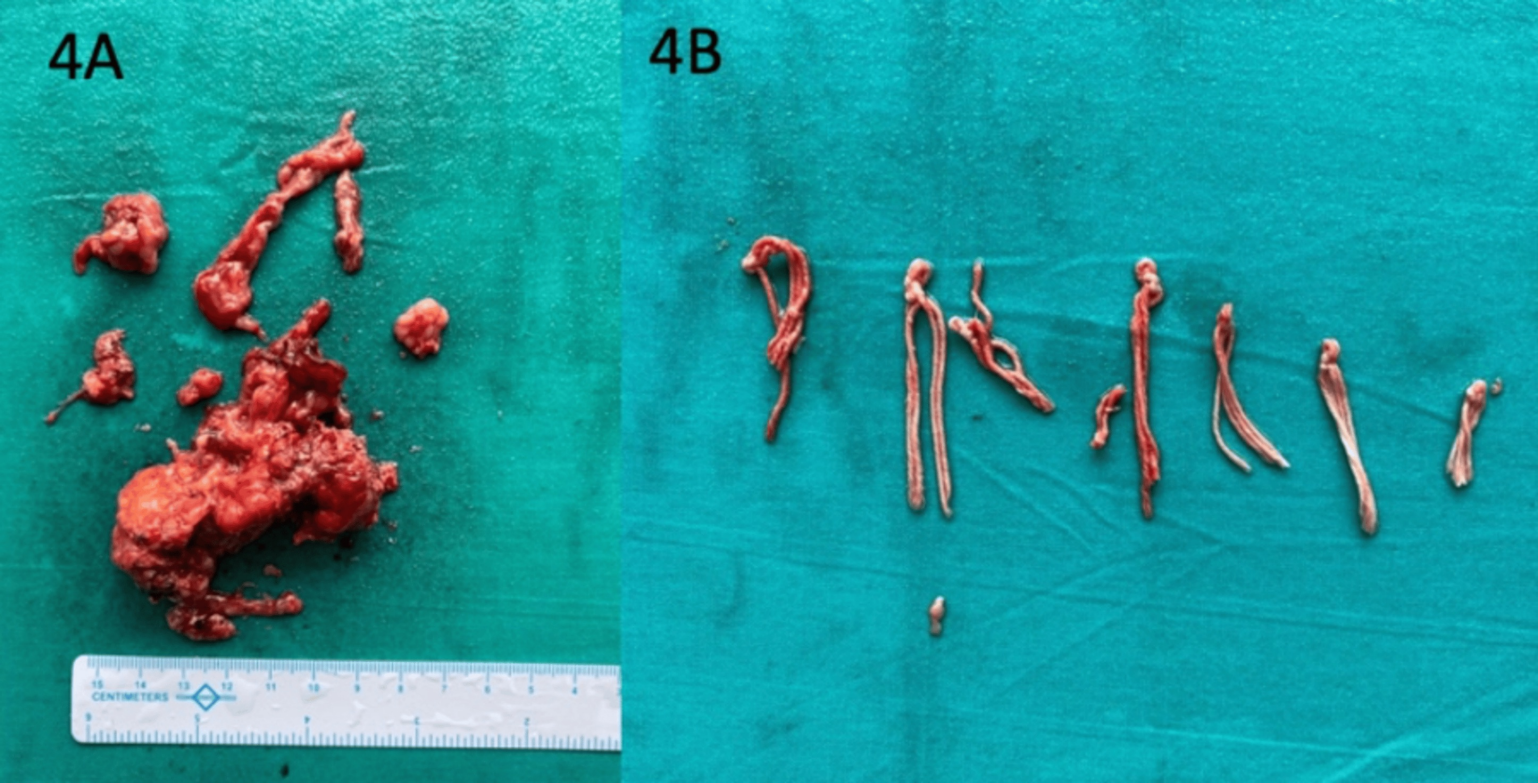
Cut section of the excised cyst with a calcified wall (a) and non-absorbable suture material (b)

The patient underwent laparoscopy cholecystectomy and the excision of the omental cyst as well. Intra-operatively about a 3x6 cm sized cyst was seen densely adherent to the anterior abdominal wall and was lying close to the transverse colon. While dissecting the omental cyst from the transverse colon and omentum, the cyst wall inadvertently ruptured, and pus-like material, along with a bundle of braided sutures, was seen coming out of the cyst (Figure 3 and Figure 4). The cyst was excised and sent for histopathological examination.

Outcome and follow-up

**Figure 5:**
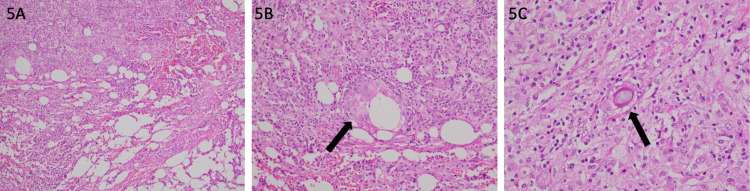
Histopathology showing inflammatory infiltrates (a), giant cells and granuloma (b), and cyst with foreign body (c)

Both the intraoperative and postoperative courses were uneventful. He was allowed oral intake from postoperative day 1 and was discharged. The patient visited the surgery follow-up clinic one month later and had no clinical complaints. The histopathology of the specimen showed chronic inflammatory changes and inflammatory granulation tissue with fat necrosis (5.2x2.5 cm) (Figure 5), along with foreign body giant cell granuloma and foamy macrophages.

## Discussion

Omental lesions are classified as solid and cystic masses. Solid lesions are classified into benign tumors such as gastrointestinal stromal tumors, leiomyoma, lipoma, and fibroma, or their malignant counterparts such as leiomyosarcoma, liposarcoma, and fibrosarcoma. Cystic lesions are classified into true omental cysts and false omental cysts (pseudocysts). True omental cysts are surrounded by a fibrous wall and are lined by epithelium. The contents of these cysts usually include serous, chylous, purulent, or rarely sanguineous fluid [3]. On the other hand, omental pseudocysts differ from true cysts in that they lack endothelial lining and a true capsule. These are less common when compared to omental and mesenteric cysts [2]. Omental pseudocysts can be classified based on their etiology into iatrogenic and non-iatrogenic. Trauma, fat necrosis, and hematoma are non-iatrogenic causes. Whereas foreign bodies such as gauze, sutures, gallstones, ventriculoperitoneal (VP) shunts, and intrauterine devices (IUDs) are the main iatrogenic causes.

The clinical presentation of omental pseudocysts and cysts is similar. While a few present with abdominal distension and vague abdominal pain, the majority of patients are asymptomatic. However, acute abdominal symptoms are seen in 11-19% of cases where torsion or rupture of the cyst has occurred [4]. In such cases, the first line of investigation is an abdominal ultrasound, which may reveal single or multiple cystic structures with thin septa, regular contours, and internal echoes reflecting their contents. Other investigations, such as CT of the abdomen, help to delineate the exact location of the cyst and its relation to the surrounding structures. An oral contrast may help to show the cyst's relation with the bowel; however, omental cysts rarely have any communication with bowel loops [5]. Finally, intravenous urography may be done if the patient presents with urinary complaints. This occurs when the cyst extends into the pelvis, which can be seen as compression of the bladder dome.

A wide variety of complications, such as ileus, bowel obstruction, bleeding, abscess, fistula formation, and biliary stone formation, have been reported with omental pseudocyst [6]. Bowel perforation due to transmural migration has been reported as well [7].

One concerning aspect of the omental pseudocyst, apart from its complications, is that radiolucent foreign bodies are difficult to diagnose preoperatively and may present as a more ominous finding. This leads to overdiagnosis and overtreatment of patients. P.N Dogra et al., in 2005, reported a case of suture foreign body granuloma masquerading as renal neoplasm. He documented that the patient presented with complaints of hematuria and flank pain. As the CT scan was suggestive of renal cell carcinoma, the patient has undergone a radical nephrectomy. However, post-operative histopathology revealed suture granuloma [8].

Ahmad Almarzouq et al., in 2014, reported a case of vesical calculus formation on a non-absorbable suture used for open inguinal hernia repair [9]. There was a case report of suture granuloma following inguinal herniorrhaphy mimicking the urachal tumor as well [10]. Similarly, we encountered a foreign body granuloma in the greater omentum around the transverse colon, forming a cyst with contents such as non-absorbable sutures and purulent fluid. The patient had a history of hernia repair 2 years prior, during which a non-absorbable suture was used and gradually migrated inside the peritoneal cavity into the greater omentum. The patient was symptomatic and had chronic vague abdominal pain refractory to medications. He underwent a diagnostic laparoscopy. Wherein, intra-operatively, a cyst with dense adhesions was found in proximity to the transverse colon.

Management of omental cysts and pseudocysts is primarily surgical. A wide range of surgical techniques are available depending on the size, location, and characteristics of the cyst. The primary treatment modality is complete enucleation of the cyst, either laparoscopic or open, and there is a low risk of recurrence following enucleation, which ranges from 0 to 13.6% [11]. These cysts are commonly connected to surrounding structures with dense adhesions. Hence, dissection and complete enucleation of the cyst are relatively tough to perform. Another concern is the cyst's proximity to the bowel. In such cases, dissection becomes a challenge, and resection of the adjacent bowel along with the cyst may be required. Bearing this in mind, this patient had laparoscopic cyst enucleation, which was meticulously done, ensuring no bowel injury.

Other management options include marsupialization and image-guided aspiration of the cyst. They are associated with a high risk of recurrence and the need for follow-up surgery due to infection and sinus formation. In addition, cysts arising from sigmoid mesocolon can prove to be challenging as cyst exposure is difficult and dissection is precarious due to the characteristic water-shed blood supply in this area. Internal drainage is one potential option; however, it too carries a significant risk of recurrence and infection, limiting its effectiveness. Therefore, enucleation is often considered the treatment of choice for such cysts [12].

The uniqueness of this case is that the omental pseudocyst is an extremely rare condition. There has been no mention of omental pseudocyst following groin hernia surgery in the English literature. The patients’ vague symptoms and the radiolucent nature of the foreign body caused a diagnostic dilemma, and its proximity to the transverse colon led to intraoperative difficulty during laparoscopy.

## Conclusions

This case brings attention to a rare but significant complication of surgical practice: the development of an omental pseudocyst caused by a retained non-absorbable suture from a previous open hernia repair. Non-absorbable sutures, while widely used in various procedures, can occasionally lead to complications that are challenging to diagnose, especially when they appear as radiolucent foreign bodies that evade detection in imaging studies like CT scans.

The patient’s vague and non-specific symptoms compounded the difficulty in reaching a diagnosis, ultimately requiring surgical intervention to address the issue. This case serves as an important reminder of the potential long-term risks associated with retained foreign materials. It emphasizes the need to counsel patients thoroughly about these risks and ensure proper documentation during the consent process.
